# A Novel Case of Metformin-Induced Pancreatitis in an Individual With Normal Dosing and No Underlying Chronic Kidney Disease

**DOI:** 10.7759/cureus.25116

**Published:** 2022-05-18

**Authors:** Anirudh R Damughatla, Sarvani Surapaneni, Anshu Wadehra, Sohaip Kabashneh, Layla Shanah

**Affiliations:** 1 Internal Medicine, Wayne State University Detroit Medical Center, Detroit, USA

**Keywords:** metformin-induced pancreatitis, acute pancreatitis, type 2 diabetes mellitus, pancreatitis, metformin

## Abstract

It is well known that most medications have side effects, and many of them have gone through years of testing with thousands of test subjects before entering the market. However, as physicians it is important to assess how patients react to the initiation of new medications not only looking for known side effects but also rare ones. Our case highlights a rare presentation of metformin-induced pancreatitis in the setting of normal renal function and appropriate dosing. We are hoping our case will create more awareness and inspire future research in exploring the pathophysiology and causes of metformin-induced pancreatitis. Moreover, we aim to make healthcare professionals mindful so that they may recognize acute pancreatitis as a side effect of metformin even in a healthy patient.

## Introduction

Acute pancreatitis is one of the most common gastrointestinal diseases with an incidence of 110 to 140 per 100,000 population [1]. The most common causes of acute pancreatitis are gallstones, alcohol, hypertriglyceridemia, and endoscopic retrograde cholangiopancreatography (ERCP) [1]. Drug-induced acute pancreatitis is a relatively rare condition accounting for only 0.1-2% of all acute pancreatitis cases with the WHO database mentioning over 500 drugs that can cause acute pancreatitis [2]. Here, we present an interesting case of a middle-aged woman who presented with abdominal pain secondary to acute pancreatitis related to metformin use. Metformin, a biguanide, is first-line therapy for non-insulin-dependent diabetes mellitus (NIDDM) patients [3]. The most common side effects of metformin include lactic acidosis, metallic taste, B12 malabsorption, and gastrointestinal side effects including nausea, vomiting, watery diarrhea, abdominal pain, anorexia, and dyspepsia [3]. A rare side effect of metformin is its association with acute pancreatitis due to overuse or renal failure [4].

## Case presentation

**Figure 1:**
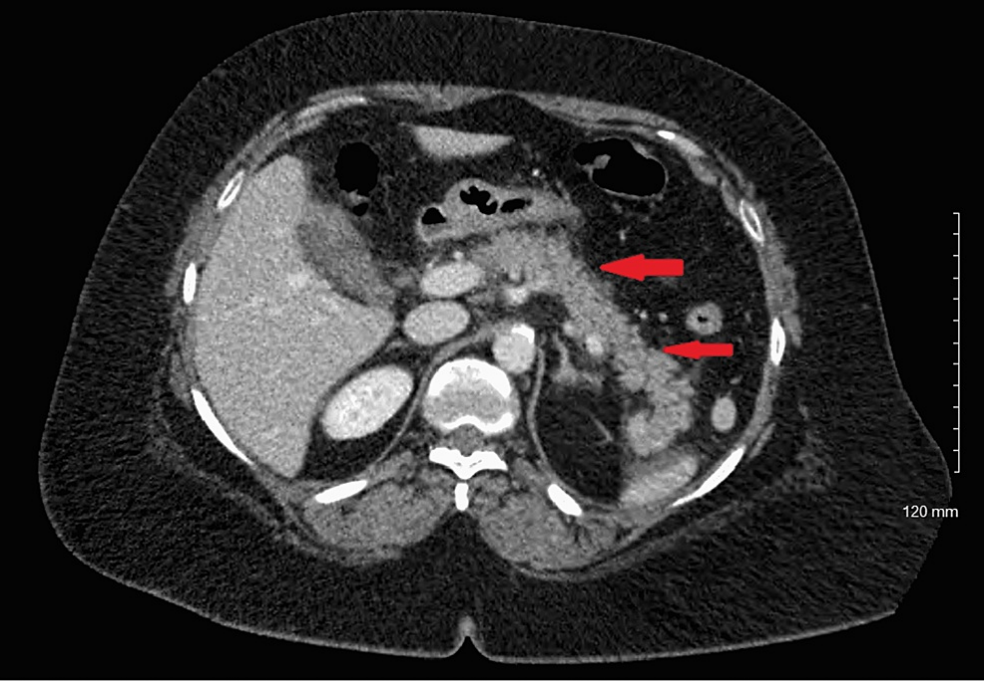
CT scan of the abdomen revealed peripancreatic effusion along the midbody and distal portion

Our patient was a 56-year-old woman with a history of hypertension and type 2 diabetes mellitus who presented with acute onset of epigastric abdominal pain and associated nausea. Physical exam was significant for epigastric tenderness to palpation and initial laboratory evaluation showed lipase levels over 6000 U/L. CT scan of the abdomen revealed a peripancreatic effusion along the midbody and distal portion (Figure 1). A diagnosis of acute pancreatitis was subsequently made.

She denied a history of alcohol use, and an ultrasound of the abdomen revealed no cholelithiasis. Triglyceride and calcium levels were within normal limits. The patient was treated with aggressive intravenous hydration and pain control. She was subsequently discharged. The etiology of this episode of acute pancreatitis could not be determined. Over the following one month, the patient was readmitted to the hospital two times with a similar clinical picture including elevated lipase levels and epigastric abdominal pain, significant for recurrent acute pancreatitis. Further imaging including magnetic resonance cholangiopancreatography (MRCP) did not show pancreatic or biliary ductal dilatation or strictures. Autoimmune workups, including IgG4 and anti-smooth muscle antibody levels, were within normal limits.

On careful medication review, it was noted that the patient was actively taking metformin 500 mg nightly, duloxetine 60 mg daily, and atenolol 25 mg daily. It was also noted that the patient was started on metformin 500 mg nightly six months before her initial admission for acute pancreatitis. During her hospitalizations, her symptoms resolved with holding metformin and recurred after resuming metformin post-discharge. Thus, metformin was held at the time of her most recent discharge and the patient did not have any recurrence of symptoms or further admissions for acute pancreatitis. A diagnosis of metformin-induced pancreatitis was thus established.

## Discussion

Metformin is one of the most widely used oral hypoglycemic agents. It is the drug of choice in the management of the majority of patients with type 2 diabetes mellitus; it is highly effective and works through several mechanisms to increase glucose transport into the cells [3]. However, like other medications, metformin is associated with side effects most notably gastrointestinal upset. Other less common, but serious side effects are lactic acidosis and acute pancreatitis [5-7].

Acute pancreatitis is one of the most frequent gastrointestinal causes of hospital admissions in the United States. In 2009 alone, it caused more than 275,000 hospitalizations in the USA [8]. Acute pancreatitis is caused by many etiologies; gallstones and alcohol abuse account for approximately two-thirds of cases [9-10]. Medications are also an established cause of acute pancreatitis. Common medications that are known to cause pancreatitis include diuretics, antimicrobial agents, HIV therapy, and neuropsychiatric agents. Metformin has been associated with pancreatitis in a few case reports.

The pathophysiology of metformin-induced pancreatitis is poorly understood, and the exact mechanism is currently unknown. Based on previously published cases, pancreatitis was caused by metformin overdose resulting in toxicity or by the use of metformin in kidney disease [6-7]. However, we report a novel case in which metformin-induced pancreatitis occurred when a standard dose was taken in a patient with intact kidney function. To our knowledge, this is the first reported case in the literature.

In our case, the patient presented multiple times with acute pancreatitis, with the constellation of findings of abdominal pain, elevated lipase, and positive CT findings. Other known causes of acute pancreatitis were negative including gallstones, alcohol abuse, hypercalcemia, and hypertriglyceridemia. In addition, the patient was not on any other medication known to cause acute pancreatitis. When calculated, the patient had a Naranjo score of 7, which is suggestive that the patient’s acute pancreatitis is probably due to the use of the metformin [11]. Once metformin was stopped as it was the only possible etiologic agent, the patient’s symptoms resolved, and she never had a recurrence of acute pancreatitis. Medication-induced pancreatitis usually has a benign course with a good prognosis. However, the lethal complications can happen without proper management, including stoppage of inciting drug [12].

## Conclusions

Our case clearly illustrates that metformin can induce pancreatitis in a healthy patient without kidney disease, even at a standard dose. Therefore, diabetic patients should be counseled about symptoms of acute pancreatitis, and the urgency to visit the emergency department and hold the medication if the symptoms occur. Healthcare professionals need to recognize acute pancreatitis as a side effect of metformin even in a healthy patient.
